# Supplementary material for: The complement C3-complement factor D-C3a receptor signalling axis regulates cardiac remodelling in right ventricular failure
Source: Nat Commun. 2022 Sep 15;13:5409. doi: 10.1038/s41467-022-33152-9 (PMC9478115; doi:10.1038/s41467-022-33152-9)
Supplement: Supplementary file 1 — Supplementary Information [file 41467_2022_33152_MOESM1_ESM.pdf]

The complement C3-complement factor D-C3a receptor signalling axis regulates cardiac remodelling in right ventricular failure

Shogo Ito[1,2], Hisayuki Hashimoto[1,3], Hiroyuki Yamakawa[1,3], Dai Kusumoto[1,3], Yohei Akiba[1], Takahiro Nakamura[1], Mizuki Momoi[1], Jin Komuro[1], Toshiomi Katsuki[1], Mai Kimura[1], Yoshikazu Kishino[1], Shin Kashimura[1], Akira Kunitomi[1], Mark Lachmann[1], Masaya Shimojima[1], Gakuto Yozu[1], Chikaaki Motoda[1], Tomohisa Seki[1,4], Tsunehisa Yamamoto[1], Yoshiki Shinya[1], Takahiro Hiraide[1], Masaharu Kataoka[1, 5], Takashi Kawakami[1], Kunimichi Suzuki[6], Kei Ito[7], Hirotaka Yada[7,8], Manabu Abe[9], Mizuko Osaka[10], Hiromi Tsuru[10], Masayuki Yoshida[10], Kenji Sakimura[9], Yoshihiro Fukumoto[2], Michisuke Yuzaki[6], Keiichi Fukuda[1], Shinsuke Yuasa[1]

[1]Department of Cardiology, Keio University School of Medicine, 35 Shinanomachi, Shinjuku-ku, Tokyo 160-8582, Japan

[2]Division of Cardio-Vascular Medicine, Department of Internal Medicine, Kurume University School of Medicine, 67 Asahi-machi, Kurume, Fukuoka 830-0011, Japan

[3]Center for Preventive Medicine, Keio University School of Medicine, 35 Shinanomachi, Shinjuku-ku, Tokyo 160-8582, Japan

[4]Department of Healthcare Information Management, The University of Tokyo Hospital, 7-3-1 Hongo, Bunkyo-ku, Tokyo 113-8655, Japan

[5]Second Department of Internal Medicine, University of Occupational and Environmental Health, 1-1 Iseigaoka, Yahatanishi-ku, Kitakyushu, Fukuoka 807-8555, Japan

[6]Department of Physiology, Keio University School of Medicine, 35 Shinanomachi, Shinjuku-ku, Tokyo 160-8582, Japan

[7]Department of Cardiology, National Defense Medical College, 3-2 Namiki, Tokorozawa, Saitama 359-8513, Japan

[8]Department of Cardiology, International University of Health and Welfare, Mita Hospital, 1-4-3 Mita Minatoku, Tokyo 108-8329, Japan

[9]Department of Animal Model Development, Brain Research Institute, Niigata University, 1-757 Asahimachi-Dori, Chuo-ku, Niigata 951-8585, Japan

[10]Department of Life Sciences and Bioethics, Graduate School of Medical and Dental Sciences, Tokyo Medical and Dental University, 1-5-45 Yushima, Bunkyo-ku, Tokyo 113-8510, Japan

Supplementary Figure 1

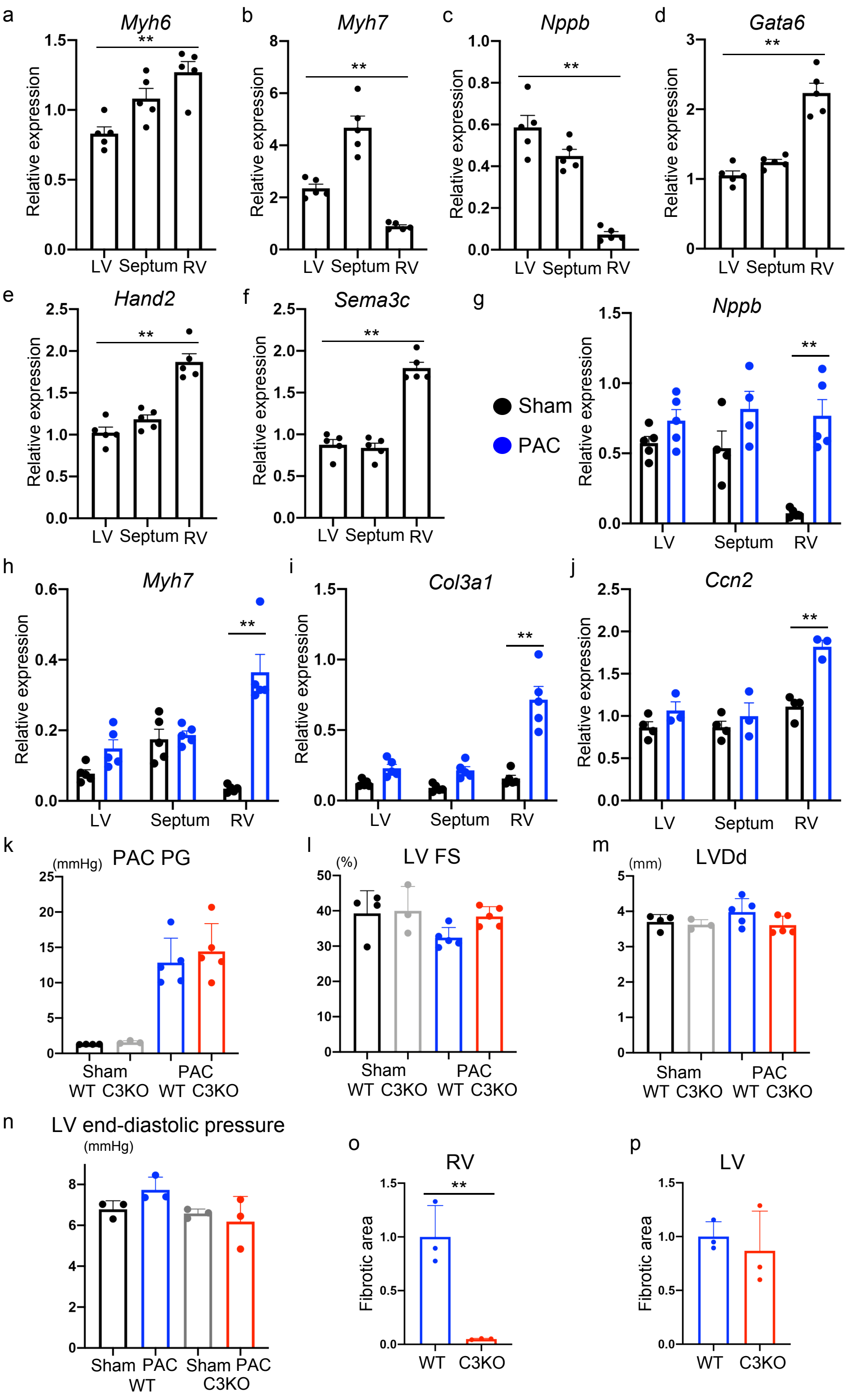

Supplementary Fig. 1: Differential gene expression patterns among different cardiac regions.

a-f, Gene expression of several cardiac markers, including *Myh6*, *Myh7*, *Nppb*, *Gata6*, *Hand2*, and *Sema3c*, were measured by qRT-PCR analysis in the left ventricle (LV), ventricular septum, and right ventricle (RV) of wild type (WT) mice (n = 5, $p = 0.0029$, $p < 0.0001$, $p < 0.0001$, $p < 0.0001$, $p = 0.0002$, $p = 0.0018$). Data are presented as mean ± standard error of the mean (SEM). **g-j,** Gene expression of several heart failure (*Nppb* and *Myh7*) and fibrotic (*Col3a1* and *Ccn2*) markers were measured by qRT-PCR analysis in the LV, ventricular septum and RV after pulmonary artery constriction (PAC) (n = 5, $p = 0.0003$, $p = 0.0002$, $p = 0.0004$, $p = 0.001$). Data are presented as mean ± SEM. In qRT-PCR analysis, expression of target genes was normalised to that of *Gapdh*. **k-m,** Measured values obtained from the echocardiogram in sham and PAC models of WT and *C3* knockout (C3KO) mice. The pressure gradient in PAC site (PAC PG), LV contractile function (left ventricular fractional shortening [LV FS]), and LV size (left ventricular end-diastolic diameter [LVDd]) were evaluated (n = 3-5). Data are presented as mean ± standard deviation. **n,** Measured values obtained from the catheter analysis in sham and PAC models of WT and C3KO mice (n = 3). LV end-diastolic pressure was evaluated. Data are presented as mean ± SD. **o-p,** Quantified fibrotic area of the RV and LV in WT and C3KO PAC model mice (n = 3, $p = 0.0049$). Data are presented as mean ± SD. Significance was assessed using a two-tailed unpaired Student's *t*-test. *$p < 0.05$; **$p < 0.01$.

Supplementary Figure 2

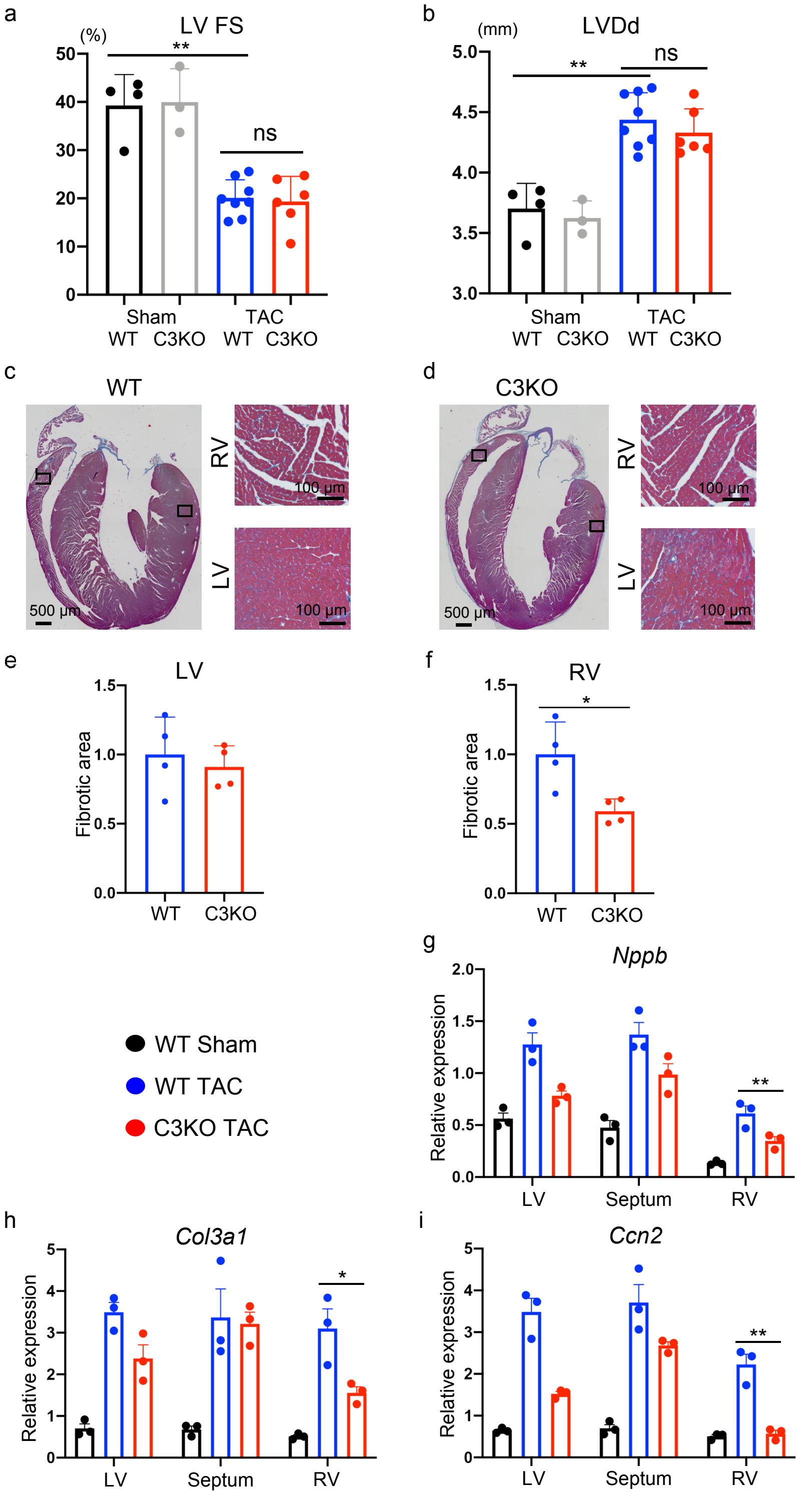

Supplementary Fig. 2: C3 deficiency does not affect left ventricular (LV) functioning in transverse aortic constriction (TAC) mouse model.

a-b, Measured values obtained from the echocardiogram of sham and TAC models in wild type (WT) and *C3* knockout (C3KO) mice (n = 4, 3, 8, 6, a; $p<0.0001$, $p = 0.762$, b; $p = 0.003$, $p = 0.370$). The LV contractile function (left ventricular fractional shortening [LV FS]) and LV size (left ventricular end-diastolic diameter [LVDd]) were evaluated. Data are presented as mean ± standard error of the mean (SEM). **c-d,** Representative images of Azan staining of the heart in WT TAC and C3KO TAC mice (n = 4). RV, right ventricle. **e, f,** Quantified fibrotic area of the LV and RV in WT TAC and C3KO TAC mice (n = 4, $p = 0.0169$). Data are presented as mean ± SEM. **g-i,** Gene expression of several heart failure (*Nppb*) and fibrotic markers (*Col3a1* and *Ccn2*) markers were measured by qRT-PCR analysis in the LV, ventricular septum, and RV of WT sham, WT TAC, and C3KO TAC mice (n = 3, $p = 0.008$, $p = 0.035$, $p = 0.003$). Data are presented as mean ± SEM. In qRT-PCR analysis, expression of target genes was normalised to that of *Gapdh*. Significance was assessed using a two-tailed unpaired Student's *t*-test. *$p < 0.05$; **$p < 0.01$; ns, non-significant.

Supplementary Figure 3

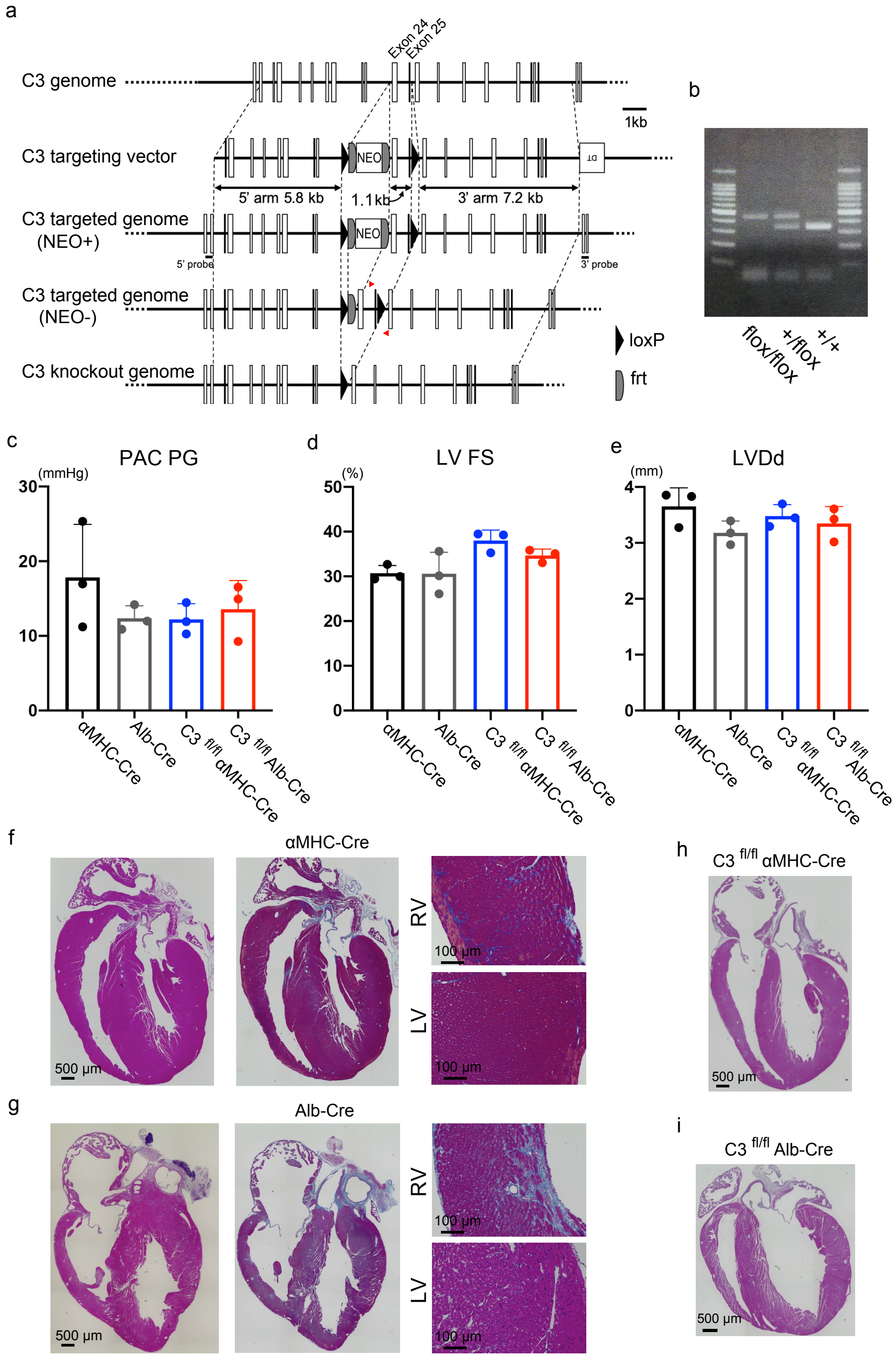

Supplementary Fig. 3: Generation of conditional *C3* knockout mice.

a, Schematic illustration of the *C3* genomic DNA, targeting vector, targeted genome, targeted genome after FLP-mediated recombination, and knockout genome after Cre-mediated recombination. Open boxes indicate exons. Two *frt* sequences (grey semicircles) were attached to remove the neomycin resistance gene (*Neo*). The black triangles indicate the *loxP* sequences. The red arrowheads indicate the primer used for genotyping. **b,** Genotyping PCR for the detection of *C3* flox/flox, +/flox, or +/+ mice (n = 3). **c-e,** Measured values obtained from the echocardiogram in α-myosin heavy chain promoter-driven Cre (αMHC-Cre) pulmonary artery constriction (PAC), and albumin promoter-driven Cre (Alb-Cre) PAC, C3 floxed αMHC-Cre ($C3^{fl/fl}$ αMHC-Cre), and C3 floxed Alb-Cre ($C3^{fl/fl}$ Alb-Cre) PAC mice (n = 3). The pressure gradient in PAC site (PAC PG), left ventricle (LV) contractile function (left ventricular fractional shortening [LV FS]), and LV size (left ventricular end-diastolic diameter [LVDd]) were evaluated. Data are presented as mean ± standard deviation. **f-g,** Representative images of hematoxylin-eosin staining and Azan staining of the heart in αMHC-Cre and Alb-Cre PAC model mice (n = 3). **h-i,** Representative images of hematoxylin-eosin staining of the heart in $C3^{fl/fl}$ αMHC-Cre and $C3^{fl/fl}$ Alb-Cre PAC model mice (n = 3).

Supplementary Figure 4

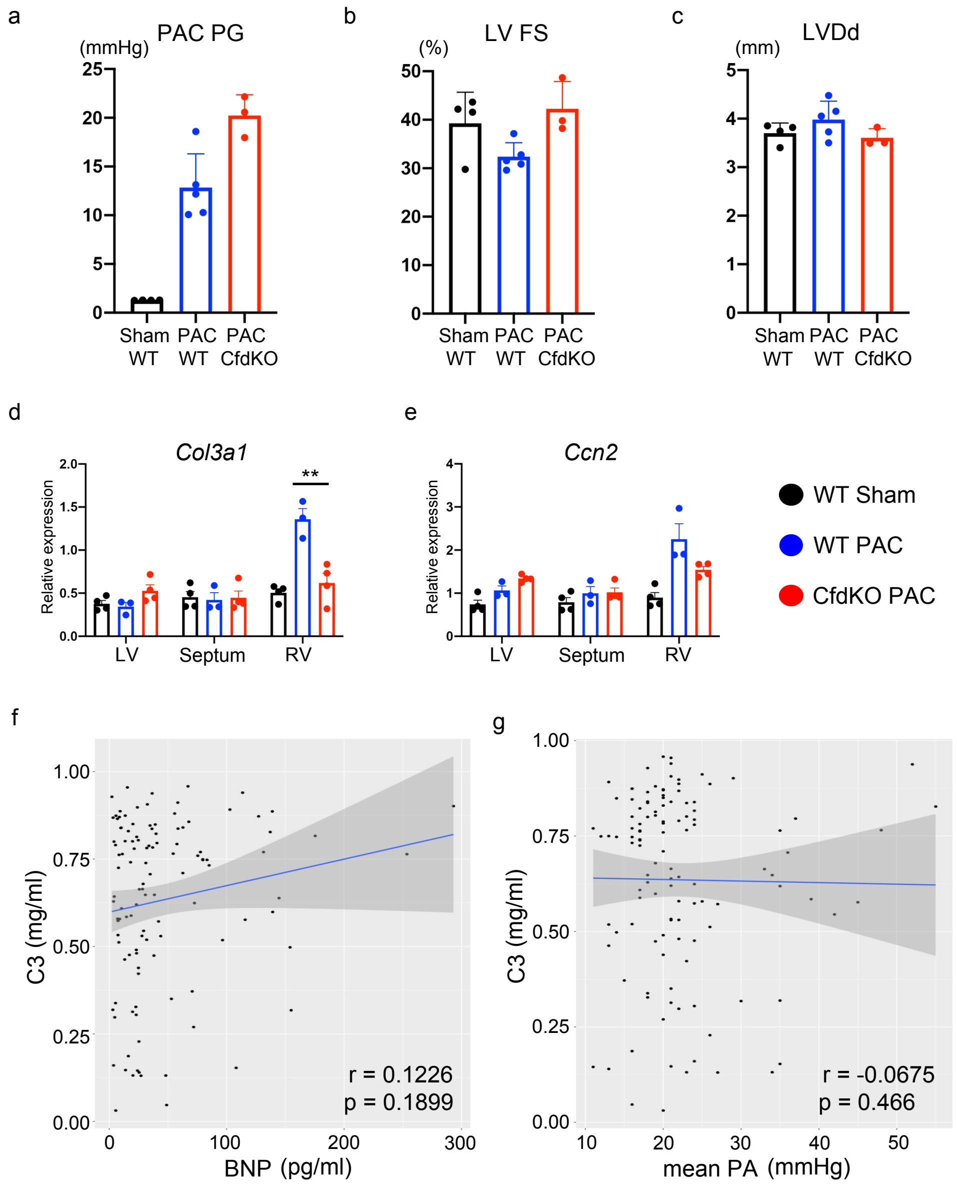

Supplementary Fig. 4: Complement factor D (Cfd) plays an important role in right ventricular (RV) failure.

a-c, Measured values obtained from the echocardiogram of wild type (WT) sham, WT pulmonary artery constriction (PAC), and *Cfd* knockout (CfdKO) PAC model mice (n = 4, 5, 3). The pressure gradient in PAC site (PAC PG), left ventricle (LV) contractile function (left ventricular fractional shortening [LV FS]), and LV size (left ventricular end-diastolic diameter [LVDd]) were evaluated. Data are presented as mean ± standard deviation. **d-e,** Gene expression of fibrotic (*Col3a1* and *Ccn2*) markers were measured by qRT-PCR analysis in the LV, ventricular septum, and RV of WT sham, WT PAC, and CfdKO PAC mice (n = 4, 3, 4, d; p = 0.007). Data are presented as mean ± standard error of the mean. In qRT-PCR analysis, expression of target genes was normalised to that of *Gapdh*. Significance was assessed using a two-tailed unpaired Student's *t*-test. *p < 0.05. **f,** Scatter plots showing the correlation between the C3 concentration and B-type natriuretic peptide (BNP) concentration in the overall cohort (n = 116; mean age = 66.7 ± 15.3 years; 70.7% women). Spearman correlation coefficient and two-tailed p-value are shown. Linear regression line (blue line) with 95% confidence intervals (gray area) is represented. **g,** Scatter plots showing the correlation between the C3 concentration and mean pulmonary artery (PA) pressure in the overall cohort (n = 119; mean age = 66.3 ± 15.5 years; 71.4% women). Spearman correlation coefficient and two-tailed p-value are shown. Linear regression line with 95% confidence intervals is represented.

Supplementary Figure 5

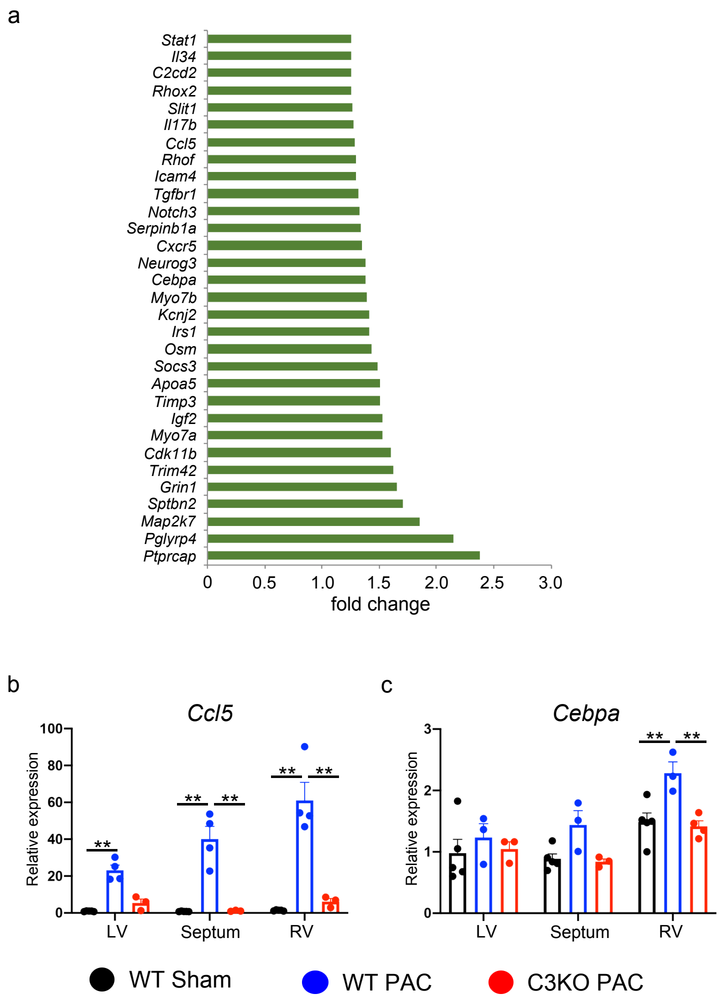

Supplementary Fig. 5: Regulation of heart failure-specific gene expression by C3a *in vitro* and *in vivo*.

a, Upregulated genes identified by global gene expression analysis of neonatal rat ventricular cardiomyocytes (NRVCs) by addition of recombinant C3a protein. **b-c,** Expression of genes upregulated *in vitro* (*Ccl5* and *Cebpa)* was measured by qRT-PCR analysis in the left ventricle (LV) ventricular septum, and right ventricle (RV) of wild type (WT) sham, WT pulmonary artery constriction (PAC), and *C3* knockout (C3KO) PAC model mice ($n = 3$-5, b; $p < 0.0001$, $p = 0.0003$, $p = 0.0051$, $p = 0.0002$, $p = 0.0055$, c; $p = 0.0017$, $p = 0.0029$). Data are presented as mean ± standard error of the mean. In qRT-PCR analysis, expression of target genes was normalised to that of *Gapdh*. Significance was assessed using a two-tailed unpaired Student's *t*-test. **$p < 0.01$.

Supplementary Figure 6

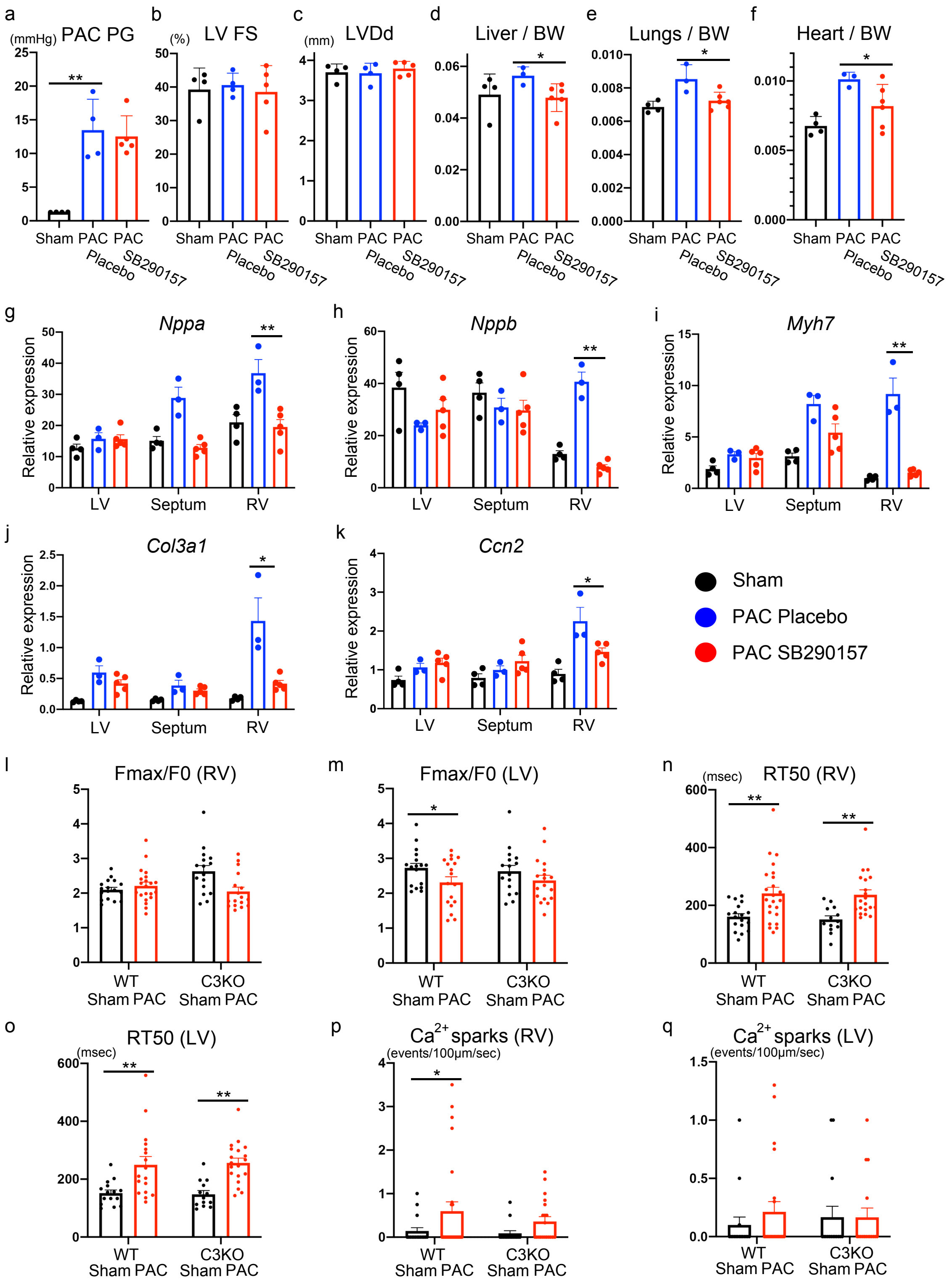

Supplementary Fig. 6: C3a receptor (C3aR) antagonist SB290157 improves right ventricular (RV) functioning after pulmonary artery constriction (PAC).

a-c, Measured values obtained from the echocardiogram in wild type (WT) sham mice, WT PAC mice under placebo, and WT PAC mice treated with SB290157 (n = 4, 4, 5, a; p = 0.0018). The pressure gradient in PAC site (PAC PG), left ventricle (LV) contractile function (left ventricular fractional shortening [LV FS]), and LV size (left ventricular end-diastolic diameter [LVDd]) were evaluated. Data are presented as mean ± standard deviation. **d-f,** Organ weight/body weight (BW) ratios in WT sham mice, WT PAC mice under placebo, and WT PAC mice treated with SB290157 (n = 4, 3, 5, d; p = 0.0440, e; p = 0.0212, f; p = 0.0143). Data are presented as mean ± standard error of the mean (SEM). **g-k,** Gene expression of several heart failure (*Nppa*, *Nppb*, and *Myh7*) and fibrotic (*Col3a1* and *Ccn2*) markers were measured by qRT-PCR analysis in the LV, ventricular septum, and RV of WT sham mice, WT PAC mice under placebo, and WT PAC mice treated with SB290157 (n = 5, 3, 5, p= 0.008, p < 0.001, p = 0.005, p = 0.011, p = 0.03). Data are presented as mean ± SEM. In qRT-PCR analysis, expression of target genes was normalised to that of *Gapdh*. **l-q,** Measured values obtained from Ca^{2+} transients in singled cardiomyocytes from RV and LV of sham and PAC models of WT and C3KO mice (n = 16, 15, 26, 20, m; p = 0.028, n; p = 0.001, p = 0.001, o; p = 0.002, p < 0.0001, p; p = 0.027). Fmax/F0, RT50, and Ca^{2+} spark frequency were evaluated. Data are presented as mean ± standard error of the mean. Significance was assessed using a two-tailed unpaired Student's *t*-test. *p < 0.05; **p < 0.01.

Table. S1| Primers for genotyping by PCR

C3KO	oIMR 1355: ATC TTG AGT GCA CCA AGC C<br>oIMR 1356: GGT TGC AGC AGT CTA TGA AGG<br>oIMR 7415: GCC AGA GGC CAC TTG TGT AG<br>Mutant = ~500 bp<br>Heterozygte = ~500 bp and 350 bp<br>Wild type = 350 bp
$C3^{flox/flox}$	C3flox-F CTCGTCCCACCTCACATAGA<br>C3flox-R GCCGCATAAAGCTGGTGCTT<br>WT: 341bp, floxed: 448bp
Cfd KO	F: ATGGGGTGGAGGGTGTTACT<br>R: ACAACAGTCCTGGGTACAGC<br>WT: ~2000bp, KO: 600bp

Table. S2| Primers for qRT-PCR

Mouse

Gapdh	F: CCAATGTGTCCGTCGTGGATCT<br>R: GTTGAAGTCGCAGGAGACAACC
C3	F: GAGCGAAGAGACCATCGTACT<br>R: TCTTTAGGAAGTCTTGCACAGTG
C3ar1	F: CTCACTTGTCTATTGGGACTGC<br>R: ATGGAGGAACCAGACTGTGTT
Cfd	F: CTACAAGCGATGGTATGATGTGC<br>R: GGACCCAACGAGGCATTCT
Nppa	F: CACAGATCTGATGGATTTCAAGA<br>R: CCTCATCTTCTACCGGCATC
Nppb	F: AGTCCTTCGGTCTCAAGGCA<br>R: CCGATCCGGTCTATCTTGTGC
Col3a1	F: CCTGGCTCAAATGGCTCAC<br>R: CAGGACTGCCGTTATTCCCG
Ccn2	F: GGGCCTCTTCTGCGATTTC<br>R: ATCCAGGCAAGTGCATTGGTA
Myh6	F: GCAGAACAGTAAAATTGAGGACG<br>R: CGCAGCTTCTCCACCTTAG
Myh7	F: GCCCTTTGACCTCAAGAAAG<br>R: CTTCACAGTCACCGTCTTGC
Ccl5	F: TGCTCCAATCTTGCAGTCGT<br>R: TCTTCTCTGGGTTGGCACAC
Cebpa	F: CAAGAACAGCAACGAGTACCG<br>R: GTCACTGGTCAACTCCAGCAC
Hand2	F: GCAGGACTCAGAGCATCAACA<br>R: AGGTAGGCGATGTATCTGGTG
Sema3c	F: GCCAGCATCAACAATCAAAGTT<br>R: TCTGAATCACCCGGACGAAAT
Gata6	F: CATCACCATCACCCGACCTAC<br>R: GGCCCTGTAAGCTGTGGAG

Human

GAPDH	F: ACAACTTTGGTATCGTGGAAGG<br>R: GCCATCACGCCACAGTTTC
C3	F: CTGTCCACGACTTCCCAGG<br>R: CCCCTTTTCTGACTTGAACTCC
CFD	F: GACACCATCGACCACGACC<br>R: GCCACGTCGCAGAGAGTTC
C3AR1	F: CCCTACGGCAGGTTCCTATG<br>R: GACAGCGATCCAGGCTAATGG

Table. S3| Lists of antibodies

**Reagent**	**Source**	**Identifier**
Mouse anti-C3d	Creative BioLabs	TAB-1360CL
Rabbit anti-p44/42 MAPK	Cell Signaling technology	4695
Rabbit anti-Phospho-p44/42 MAPK	Cell Signaling technology	4370
Rabbit anti-Phospho-p38	Cell Signaling technology	9211
Rabbit anti-p38	Cell Signaling technology	9212
Rabbit anti-SAPK/JNK	Cell Signaling technology	9252
Rabbit anti-phospho-SAPK/JNK	Cell Signaling technology	9251

Table. S4| Lists of chemicals, recombinant proteins, and siRNA

**Reagent**	**Source**	**Identifier**
Recombinant Mouse Complement Component C3a	R&D Systems	8085-C3-025
SB290157	Sigma Aldrich	SML1192-25MG
Endthelin 1	Sigma Aldrich	E7764-1MG
C3aR1 siRNA	Ambion	s136363 Cat:4390771
