# Supplementary material for: The complement C3-complement factor D-C3a receptor signalling axis regulates cardiac remodelling in right ventricular failure
Source: Nat Commun. 2022 Sep 15;13:5409. doi: 10.1038/s41467-022-33152-9 (PMC9478115; doi:10.1038/s41467-022-33152-9)
Supplement: Supplementary file 3 — Reporting Summary [file 41467_2022_33152_MOESM3_ESM.pdf]

nature portfolio

Corresponding author(s): Shinsuke Yuasa

Last updated by author(s): Jun 22, 2022

Reporting Summary

Nature Portfolio wishes to improve the reproducibility of the work that we publish. This form provides structure for consistency and transparency in reporting. For further information on Nature Portfolio policies, see our Editorial Policies and the Editorial Policy Checklist.

Statistics

For all statistical analyses, confirm that the following items are present in the figure legend, table legend, main text, or Methods section.

n/a	Confirmed	
☐	☒	The exact sample size (n) for each experimental group/condition, given as a discrete number and unit of measurement
☐	☒	A statement on whether measurements were taken from distinct samples or whether the same sample was measured repeatedly
☐	☒	The statistical test(s) used AND whether they are one- or two-sided<br>*Only common tests should be described solely by name; describe more complex techniques in the Methods section.*
☐	☒	A description of all covariates tested
☐	☒	A description of any assumptions or corrections, such as tests of normality and adjustment for multiple comparisons
☐	☒	A full description of the statistical parameters including central tendency (e.g. means) or other basic estimates (e.g. regression coefficient) AND variation (e.g. standard deviation) or associated estimates of uncertainty (e.g. confidence intervals)
☐	☒	For null hypothesis testing, the test statistic (e.g. F, t, r) with confidence intervals, effect sizes, degrees of freedom and P value noted<br>*Give P values as exact values whenever suitable.*
☒	☐	For Bayesian analysis, information on the choice of priors and Markov chain Monte Carlo settings
☒	☐	For hierarchical and complex designs, identification of the appropriate level for tests and full reporting of outcomes
☒	☐	Estimates of effect sizes (e.g. Cohen's d, Pearson's r), indicating how they were calculated

Our web collection on statistics for biologists contains articles on many of the points above.

Software and code

Policy information about availability of computer code

Data collection	Transcriptome analysis was performed using SurePrint G3 Mouse Microarray 8X60K ver.2.0 (Agilent Technologies Inc., Santa Clara, CA, USA) and SurePrint G3 Rat GE 8 × 60K ver. 2.0 (Agilent Technologies Inc.). The chips were scanned using Agilent Scanner G2505C (Agilent Technologies Inc.). The fibrotic area was determined using the ImageJ software (https://imagej.nih.gov/ij/download.html).
Data analysis	The data were processed and analysed using the GeneSpring software (v.14.1.1, Agilent Technologies Inc.).

For manuscripts utilizing custom algorithms or software that are central to the research but not yet described in published literature, software must be made available to editors and reviewers. We strongly encourage code deposition in a community repository (e.g. GitHub). See the Nature Portfolio guidelines for submitting code & software for further information.

Data

Policy information about availability of data

All manuscripts must include a data availability statement. This statement should provide the following information, where applicable:

- Accession codes, unique identifiers, or web links for publicly available datasets
- A description of any restrictions on data availability
- For clinical datasets or third party data, please ensure that the statement adheres to our policy

The microarray data obtained in this study have been deposited in the GEO database with the accession code GSE183503 and GSE183504. All the other data supporting the findings of this study are available within the article and its Supplementary Information files. Source data are provided with this paper.

Field-specific reporting

Please select the one below that is the best fit for your research. If you are not sure, read the appropriate sections before making your selection.

☒ Life sciences ☐ Behavioural & social sciences ☐ Ecological, evolutionary & environmental sciences

For a reference copy of the document with all sections, see nature.com/documents/nr-reporting-summary-flat.pdf

Life sciences study design

All studies must disclose on these points even when the disclosure is negative.

Sample size	Sample size was chosen as a result of previous experience regarding data variability in similar models and experiments. The determination of sample size and data analysis for this study followed the general guideline for animal studies (https://pubmed.ncbi.nlm.nih.gov/12391400/). No statistical method was used to predetermine sample size.
Data exclusions	No data were excluded from the analyses.
Replication	All experiments except for human sample RT-PCR analysis were carried out in at least 3 biological replicates. The exact number of biological replicates (number of mice, samples or cell culture dishes) is mentioned in the Figure legends. Human sample RT-PCR analysis was carried out in technical independent 3 replicates due to the sample limitation.
Randomization	The samples used in this study were randomly assigned to control or experimental groups.
Blinding	The investigators were blinded for mouse genotype and treatment during surgeries, echocardiography, cardiac catheterization, organ weight determination and all histological and immunofluorescence quantifications. There was no blinding for in vitro cellular experiment. The investigators were blinded to group allocation during data collection and/or analysis. For the analysis of human complement measurements, the investigators are also blinded.

Reporting for specific materials, systems and methods

We require information from authors about some types of materials, experimental systems and methods used in many studies. Here, indicate whether each material, system or method listed is relevant to your study. If you are not sure if a list item applies to your research, read the appropriate section before selecting a response.

Materials & experimental systems

n/a	Involved in the study	
☐	☒	Antibodies
☒	☐	Eukaryotic cell lines
☒	☐	Palaeontology and archaeology
☐	☒	Animals and other organisms
☐	☒	Human research participants
☐	☒	Clinical data
☒	☐	Dual use research of concern

Methods

n/a	Involved in the study	
☒	☐	ChIP-seq
☒	☐	Flow cytometry
☒	☐	MRI-based neuroimaging

Antibodies

Antibodies used	The following primary antibodies were used for immunohistological staining:<br>Mouse anti-C3d (clone 3d29) (#TAB-1360CL, 1:100, Creative Biolabs)<br>Anti-Mouse-IgG-HRP (#NA931, 1:200, GE Healthcare Life Sciences)<br><br>The following primary antibodies were used for western blotting analysis:<br>Rabbit anti-p44/42 MAPK (#4695, 1:1000, Cell Signaling technology)<br>Rabbit anti-Phospho-p44/42 MAPK (#4370, 1:1000, Cell Signaling technology)<br>Rabbit anti-Phospho-p38 (#9211, 1:1000, Cell Signaling technology)<br>Rabbit anti-p38 (#9212, 1:1000, Cell Signaling technology)<br>Rabbit anti-SAPK/JNK (#9252, 1:1000, Cell Signaling technology)<br>Rabbit anti-Phospho-SAPK/JNK (#9251, 1:1000, Cell Signaling technology)<br>Anti-Rabbit-IgG-HRP (#NA934, 1:2000, GE Healthcare Life Sciences)
Validation	Anti-C3d (clone 3d29) (#TAB-1360CL, Creative Biolabs) has been validated in the previous report (J Clin Invest. 2013 May 1; 123(5): 2218–2230.).<br>Anti-p44/42 MAPK (#4695, Cell Signaling technology): Validation was stated on the manufacturer's website and confirmed for WB.<br>Anti-Phospho-p44/42 MAPK (#4370, Cell Signaling technology): Validation was stated on the manufacturer's website and confirmed

for WB.
Anti-Phospho-p38 (#9211, Cell Signaling technology): Validation was stated on the manufacturer's website and confirmed for WB.
Anti-p38 (#9212, Cell Signaling technology): Validation was stated on the manufacturer's website and confirmed for WB.
Anti-SAPK/JNK (#9251, Cell Signaling technology): Validation was stated on the manufacturer's website and confirmed for WB.
Anti-Phospho-SAPK/JNK (#9252, Cell Signaling technology): Validation was stated on the manufacturer's website and confirmed for WB.
Anti-Mouse-IgG-HRP (#NA931, GE Healthcare Life Sciences): Validation was stated on the manufacturer's website and confirmed for IHC.
Anti-Rabbit-IgG-HRP (#NA934, GE Healthcare Life Sciences): Validation was stated on the manufacturer's website and confirmed for WB.

Animals and other organisms

Policy information about studies involving animals; ARRIVE guidelines recommended for reporting animal research

Laboratory animals	For all mice experiments, male mice at 8-10 weeks were used at sham, pulmonary artery constriction or transverse aortic constriction operation. After 2-4 weeks after surgery, operated mice were examined for the subsequent analyses.<br>The animals had free access to water and a standard diet and were maintained on a 12-h light and dark cycle at a room temperature of 22 ± 2 °C and a humidity of 35–60%. For in vitro cardiomyocyte study, neonatal rat ventricular cardiomyocytes were prepared for primary culture.
Wild animals	The study did not involve wild animals.
Field-collected samples	No field-collected samples were used.
Ethics oversight	Experiments in this study were approved by Animal Ethics Committee at Keio University.

Note that full information on the approval of the study protocol must also be provided in the manuscript.

Human research participants

Policy information about studies involving human research participants

Population characteristics	133 patients, including 128 with chronic thromboembolic pulmonary hypertension (CTEPH) and 5 with Pulmonary arterial hypertension (PAH), were enrolled for the main analysis (mean age=66.1 ± 15.3 years; 70.7% women).
Recruitment	We retrospectively evaluated patients with CTEPH and PAH from April 2016 to November 2020 according to our inclusion criteria: (1) age ≥18 years, (2) subjected to right heart catheterisation (RHC), and (3) availability of plasma samples.
Ethics oversight	The present study was approved by the Ethics Committee of Keio University Hospital (approval no. 20140203), and informed consent was obtained from all participants.

Note that full information on the approval of the study protocol must also be provided in the manuscript.

Clinical data

Policy information about clinical studies

All manuscripts should comply with the ICMJEguidelines for publication of clinical research and a completedCONSORT checklist must be included with all submissions.

Clinical trial registration	*Provide the trial registration number from ClinicalTrials.gov or an equivalent agency.*
Study protocol	*Note where the full trial protocol can be accessed OR if not available, explain why.*
Data collection	*Describe the settings and locales of data collection, noting the time periods of recruitment and data collection.*
Outcomes	*Describe how you pre-defined primary and secondary outcome measures and how you assessed these measures.*
